# Primary hyperoxaluria in Italy: the past 30 years and the near future of a (not so) rare disease

**DOI:** 10.1007/s40620-022-01258-4

**Published:** 2022-02-26

**Authors:** Giorgia Mandrile, Alessandra Pelle, Veronica Sciannameo, Elisa Benetti, Maria Michela D’Alessandro, Francesco Emma, Giovanni Montini, Licia Peruzzi, Michele Petrarulo, Renato Romagnoli, Corrado Vitale, Barbara Cellini, Daniela Giachino

**Affiliations:** 1 Genetic Unit and Thalassemia Center, San Luigi Gonzaga University Hospital, Regione Gonzole 10, 10043 Orbassano, TO Italy; 2 Medical Genetics Unit, AOU Città della Salute e della Scienza, Turin, Italy; 3 Unit of Biostatistics, Epidemiology and Public Health, Department of Cardiac, Thoracic, Vascular Sciences and Public Health, University of Padova, Padua, Italy; 4 Pediatric Nephrology, Dialysis and Transplant Unit, Department of Women’s and Children’s Health, Padua University Hospital, Padua, Italy; 5 Pediatric Nephrology Unit, Ospedale dei Bambini, A.R.N.A.S. Civico-G. Di Cristina, Benfratelli Palermo, PA Italy; 6 Division of Nephrology, Department of Pediatric Subspecialties, Bambino Gesù Children’s Hospital-IRCCS, Rome, Italy; 7 Pediatric Nephrology, Dialysis and Transplant Unit, Fondazione IRCCS Ca’ Granda, Ospedale Maggiore Policlinico, Milano, Italy; 8 Department of Clinical Sciences and Community Health, University of Milan, Milan, Italy; 9 Pediatric Nephrology Unit, “Regina Margherita Department of Children’s Diseases”, Città della Salute e della Scienza di Torino, Turin, Italy; 10 Kidney Stone Laboratory-Chemical-Clinical Laboratory Unit, Azienda Ospedaliera Ordine Mauriziano di Torino, Turin, Italy; 11 Liver Transplant Unit, General Surgery 2U, Azienda Ospedaliera Universitaria Città della Salute e della Scienza di Torino, University of Turin, Turin, Italy; 12 Nephrology and Dialysis Unit, Azienda Ospedaliera Ordine Mauriziano di Torino, Turin, Italy; 13 Department of Medicine and Surgery, University of Perugia, Perugia, Italy; 14 Medical Genetic Unit, San Luigi Gonzaga University Hospital, Orbassano, TO Italy; 15 Medical Genetics, Department Clinical and Biological Sciences, University of Torino, Turin, Italy

**Keywords:** Primary hyperoxaluria, Italy, Genotype–phenotype

## Abstract

**Background:**

Primary hyperoxalurias (PHs) are rare autosomal recessive diseases of the glyoxylate metabolism; PH1 is caused by mutations in the *AGXT* gene, PH2 in *GRHPR* and PH3 in *HOGA1*.

**Methods:**

Here we report the first large multi-center cohort of Italian PH patients collected over 30 years (1992–2020 median follow-up time 8.5 years). Complete genotype was available for 94/95 PH1 patients and for all PH2 (n = 3) and PH3 (n = 5) patients. Symptoms at onset were mainly nephrolithiasis (46.3%) and nephrocalcinosis (33.7%). Median age at onset of symptoms and diagnosis were 4.0 years and 9.9 years, respectively.

**Results:**

Fifty-four patients (56.8%) were diagnosed after chronic kidney disease. Sixty-three patients (66.3%) developed end stage kidney disease (median age 14.0 years). Twenty-one patients had a kidney-only transplant and, among them, seven had a second kidney transplant combined with liver transplant. A combined kidney–liver transplant was carried out in 29 patients and a sequential kidney–liver transplant was performed in two. In five cases a preemptive liver transplant was performed. Those receiving a liver-only transplant tended to have lower kidney function at last follow-up.

**Conclusion:**

Our study of PHs in Italy underlines a considerable diagnostic delay, which has only slightly decreased in recent years. Therefore, we suggest a more extensive use of both metabolic screening among patients with recurrent kidney stones and genotyping, including unambiguous assignment of minor/major allele status in order to promptly begin appropriate treatment. This will be fundamental in order to have access to the new therapies, which are mainly focused on substrate reduction for the oxalate-producing enzymes using RNA-interference.

**Graphical abstract:**

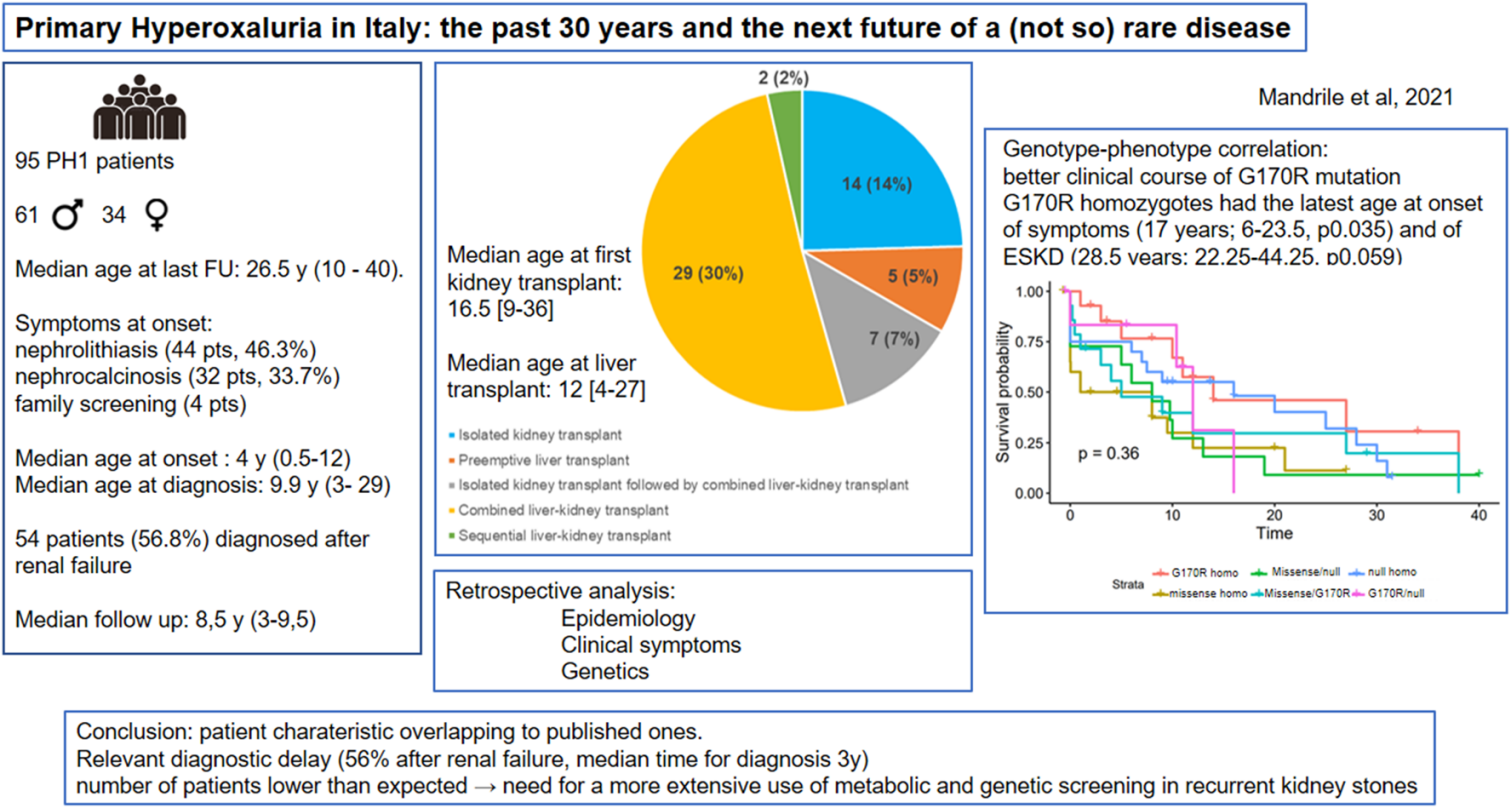

**Supplementary Information:**

The online version contains supplementary material available at 10.1007/s40620-022-01258-4.

## Background

Primary hyperoxalurias (PHs) are a group of rare autosomal recessive diseases driven by alterations in the glyoxylate metabolism and characterized by genetic heterogeneity and variable clinical presentation [1]. PH1 is estimated to occur in 1:120,000 live births in Europe [2]. Clinical cohorts evaluate a prevalence ranging from 1:1,000,000 to 3:1,000,000 depending on the population studied [2] but population analysis suggests that Primary hyperoxalurias are an order of magnitude more common than determined in clinical cohorts. Summary data obtained from whole genome projects indicate an overall carrier frequency and an inferred prevalence of PHs of 1:70 and 1:58,000, respectively [3]. It is estimated that Primary hyperoxalurias occur in 7–14% of children with nephrocalcinosis and represent the cause of renal replacement therapy before the age of 15 years in approximately 2% of patients in European and North American countries [1]. Clinical signs range from severe nephrocalcinosis in the first years of life and progression to diffuse multisystem oxalosis, to recurrent urolithiasis in adult patients [1].

PH type 1 (PH1), with an overall estimated frequency of 1:151,000 (carrier frequency 1:195) [3], is the most common and severe form, eventually leading the majority of patients to end-stage kidney disease (ESKD). PH1 is caused by mutations in the *AGXT* gene, encoding for the enzyme alanine:glyoxylate aminotransferase (AGT). AGT catalyzes the transamination of alanine and glyoxylate to pyruvate and glycine in the peroxisomal matrix of hepatocytes [4]. *AGXT* has two common intragenic haplotypes, the Major (Ma) and the minor (mi) alleles (frequency of 80% and 20%, respectively in the Caucasian population) [5]. Although not pathogenic per se, the mi allele enhances the deleterious effects of several common PH1 mutations [6–8]. Among Caucasians, the most common PH1 mutation, accounting for approximately 30–40% of alleles, is the p.Gly170Arg (G170R) in the background of the mi haplotype. This variant is characterized by enzyme instability and mistargeting to mitochondria [9], 10. Other recurrent mutations are c.33dupC (11%) on the Ma haplotype, and p.Ile244Thr (I244T) (6%) which is present in patients of Spanish or North African origin [11]. Some mutations, such as p.Gly170Arg (G170R) and p.Phe152Ile (F152I), are responsive to pyridoxine (vitamin B6–VB6) supplementation, but at present, the only effective treatment is liver- or combined kidney/liver transplantation [12].

PH type 2 (PH2) is caused by mutations in the gene glyoxylate reductase/hydroxypyruvate reductase (*GRHPR*), encoding the enzyme responsible for the reduction of glyoxylate to glycolate and hydroxypyruvate to D-glycerate. PH2 overall estimated frequency is 1:310,000 (carrier frequency 1:279) [3] and accounts for 10% of PH cases [13]. Most PH2-associated mutations produce an aberrant protein or affect catalytic activity [14]. Initially considered milder than PH1, a large cohort study recently published by Garrelfs et al. has instead shown kidney impairment in 50% of patients, of whom 25% are in ESKD [15].

PH type 3 (PH3), due to mutations in the 4-hydroxy-2-oxoglutarate aldolase 1 (*HOGA1*) gene (formerly known as *DHDPLS*), is considered the mildest form of PH. HOGA1 is responsible for the conversion of 4-hydroxy-oxoglutarate to pyruvate and glyoxylate in mitochondria. PH3 accounts for 8–10% of PHs [13] and is characterized by recurrent nephrolithiasis, in some cases accompanied by hypercalciuria, that usually decreases with age [16, 17]. Kidney function is preserved in most patients, although progressive impairment over decades is observed [13] and Martin-Higueras et al. reported that 21.4% of patients had chronic kidney disease (CKD) stages 2 or higher [18]. PH3 overall estimated frequency is 1:135,000 (carrier frequency 1:185) [3].

The Italian study of Primary hyperoxaluria started in the 1980s and the first reports of *AGXT* analysis date back to 1998–1999 [19, 20]. In 2008 several clinicians involved in PHs jointly founded OXALEurope, the European Hyperoxaluria Consortium (www.OxalEurope.org), in order to promote a continuous exchange of data, procedures and knowledge among European colleagues, with the main objective of providing PH patients the same high-level standard of care. With the beginning of PH genetic analysis in Italy, efforts were made to collect the clinical data of Italian patients and the aim of this paper is to present the state of the art of PH in Italy.

## Methods

### Patients

PH patients were recruited from January 1992 to July 31st, 2020; the cohort includes 95 PH1 patients (61 males, 64.2%), 3 PH2 and 5 PH3 patients (Table 1) [IRB approval May 20th, 2014, no. 8616, updated on April 26th, 2021, no. 6492, San Luigi Hospital]. The suspicion of primary hyperoxaluria was based on the patient’s history (recurrent or juvenile nephrolithiasis, nephrocalcinosis, ESKD without other causes, kidney biopsy with oxalate deposition); diagnosis workup started with biochemical tests, and genetic analysis was required for diagnosis confirmation. Genotype data were available for 94 out of 95 PH1 patients and for all PH2 (n = 3) and PH3 (n = 5) patients. *AGXT* analysis was performed by DHPLC until 2009 and by Sanger sequencing of the entire coding region thereafter; MLPA analysis was performed to confirm deletion [21]; the minor/Major haplotype was assigned to each patient. *GRHPR* analysis was set up in 2007 and *HOGA1* in 2012 by Sanger sequencing of the entire coding region [22]. The diagnostic workup began, in most patients, with oxalate dosage measurement in urine or plasma (in patients with an eGFR of 30–45 ml per min per 1.73 m^2^ urinary oxalate is not reliable [2]), or liver biopsy, followed by confirmatory genetic testing. Two cases were diagnosed by kidney biopsy after a transplant failure. Pyridoxine responsiveness was recorded according to the clinician’s report. Urinary oxalate results were not included for several patients due to difficulties in harmonizing the data obtained in different laboratories. For patients who were included, biochemical analyses on urine and plasma were performed by gas chromatography. Follow-up time was measured from the onset of symptoms to the last clinical visit or death.

**Table 1:** Clinical characteristics of the Italian PH population

	PH1	PH1 missing data (%)	PH2	PH3
*n*	95		3	5
Males (%)	61 (64.2)		2 (66)	3 (60)
Females (%)	34 (35.8)		1 (34)	2 (40)
Ethnicity				
Caucasian	92 (97)		3 (100)	5 (100)
South Asian	3 (3)			
Deceased	19 (20)		0	0
Age at death—years (median [IQR])	37.0 [19.0, 61.0]			
Age at onset of symptoms—years (median [IQR])	4.0 [0.5, 12.0]	6.3	13.0 [11.0, 15.0]	2.0 [2.0, 2.0]
Age at diagnosis—years (median [IQR])	9.9 [3.1, 29.0]	5.3	28.0 [23.0, 32.0]	5.5 [5.5, 11.0]
Time for diagnosis—years (median [IQR])	3.1 [0.25, 10.0]	6.3	13.0 [7.5, 18.5]	4.0 [4.0, 8.0]
Age at last follow up (median [IQR])	26.5 [10.0, 40.8]	1.1	25.0 [14.5, 40.5]	14.0 [14.0, 16.0]
Follow up years—years (median [IQR])	8.5 (3.1–9.5)		6.0 [5.0, 10.0]	1.0 [1.0, 5.0]
Parental consanguinity (%)	15 (15.8)	18.9	0	0
Symptoms at onset (%)		2.1		
Other affected family members	4 (4.2)	2		
Hematuria	1 (1.1)		0	0
Acute kidney injury	1 (1.1)		0	0
Chronic kidney disease	3 (3.2)		0	0
Nephrocalcinosis (NC)	32 (33.7)		1	0
Nephrolithiasis (NL)	44 (46.3)		2	5
NC + NL	8 (8.4)		0	0
Test requested because of other affected family member	4 (4.2)		0	0
Diagnosis after chronic kidney disease (%)—total cohort	54 (56.8)	9	0	0
Patient age at onset 0–3 years	23 (53.5)	14.0		
Patient age at onset 3–16 years	16 (55.2)	3.4		
Patient age at onset > 16 years	14 (82.4)	5.9		
Patients who experienced ESKD	63 (66.3%)		1 (34)	0
Age at ESKD—years (median [IQR])—total cohort	14.0 [5.0, 31.5]	33.7	48	
Patient age at onset 0–3 years	0.7 [0.3–10.0]			
Patient age at onset 3–16 years	23.0 [14.0–33.0]			
Patient age at onset > 16 years	40.0 [31.0–50.0]			
eGFR at last follow up (ml/min/1.73 m^2^)^a^ mean (SD)	64.2 (32.9)	56.8	94 (53)	76 (20)
Transplant (%)				
Isolated kidney transplant	14 (14.7)		1	0
Preemptive liver transplant	5 (5)		0	0
Isolated kidney transplant followed by combined liver–kidney transplant	7 (7)		0	0
Combined liver–kidney transplant	29 (30.5)		0	0
Sequential liver–kidney transplant	2 (2)		0	0
Age at first kidney transplant—years (median [IQR])	16.5 [9.3, 36.3]	43.2	52 (1 patient)	
Age at second kidney transplant—years (median (IQR))	29.0 (17.0–45.0)	83.2		
Age at liver transplant—years (median [IQR])	12.0 [4.0, 27.0]	52.6		
Pyridoxine response (%)		36.8		
No	40 (42.1)			
Yes	20 (21.1)			
Type of mutation (%)				
Homozygotes for a missense mutation (M/M)	22 (23)			
Homozygotes for a null mutation (N/N)	21 (22)			
G170R homozygotes (G170R/G170R)	16 (16.8)			
Compound heterozygotes M/N	12 (12.6)			
Compound heterozygotes M/G170R	14 (14.7)			
Compound heterozygotes N/G170R	9 (9.5)			

^a^eGFR was calculated by CKD-EPI in adults and by Schwartz equation in pediatric patients

Missing data: percentages are indicated; empty cells indicate that no data are missing

### Statistical analysis

Continuous variables were expressed as median and I-III quartiles as measures of variability; categorical variables were expressed as percentages and absolute numbers. For patients affected by PH1, renal survival was calculated by the Kaplan–Meier method in a multivariate analysis adjusted for age and gender, with patient data censored at last follow-up. The effect of different genotype classes on time to ESKD was estimated using Cox proportional hazard models with adjustment for gender. Proportional hazard assumption was checked using the Grambsch and Therneau test. Continuous variables were expressed as median and I–III quartiles as measures of variability; categorical variables were expressed as percentages and absolute numbers. F test was used to evaluate clinical variables among genotype groups. Median times to ESKD and to symptoms were computed for each genotype using the Kaplan–Meier method and differences were assessed by the log-rank test. All statistical testing was conducted at the 0.05 level. PASW Statistics (version 20) and R (version 2.13) statistical packages were used for analysis. In the genotype–phenotype analysis we grouped *AGXT* mutations into three classes according to the expected effect on the protein product, as previously published [23]:

1. G170R (T): associated with mitochondrial mistargeting when *in cis* with the minor allele [9]
2. Other missense (M): missense and in frame ins/del that are unlikely to cause complete lack of protein function.
3. Null (N): exonic deletion, nonsense, frameshift and splice-site mutations, assumed to produce no protein product.

## Results

### Population characteristics

Median age of the PH1 cohort at last follow up was 26.5 years (I–III quartile: 10.0, 40.8). Nineteen patients (20%) were deceased at the time of analysis. Median age at death was 37.0 years (19.0–61.0). Thirteen patients were lost to follow-up (14%). Fifty-one patients underwent at least one clinical examination after January 2018. Fifteen patients were born from consanguineous parents (information not available for 18 patients). In 36 cases, a family history of kidney stones was reported. Symptoms at onset were mainly nephrolithiasis (NL, 44 patients, 46.3%) and nephrocalcinosis (NC, 32 patients, 33.7%), consistent with the clinical presentation of PH patients. Four cases were diagnosed at family screening before the onset of symptoms. Median age at onset and diagnosis were 4.0 years (0.5–12.0) and 9.9 years (3.1–29.0), respectively. Fifty-four patients (56.8%) were diagnosed after renal failure. Median time to diagnosis was 3.2 years (0.3–10.0). A reduced time to diagnosis was observed for patients diagnosed more recently (after 2010), while no differences were observed if the age at onset of symptoms was considered.

### Biochemical analysis

Median plasma oxalate at diagnosis was 130 μmol/L (24.0–187.0; data not available for 23 patients) and median urinary oxalate was 230 μM/mM creat/24 h (151.0–435.5; data not available for 41 patients). Data regarding glycolate dosage was only available for a small number of patients as it is no longer evaluated in most Italian hospitals. Median plasma glycolate data, available for 27 patients, was 183 μmol/L (125.5–366.0) and median urinary glycolate data, available for 20 patients, was 319 μM/mM creat/24 h (184.5–463.0). Before being replaced by genetic testing (i.e. in the past 15 years), liver biopsy for AGT activity measurement had been performed in 35 of the study patients. Mean residual AGT activity was negligible in 27 out of 35 patients and partial AGT activity was detected in the remaining ones (mean activity: 39 ± 13% of the normal AGT activity). The response to VB6 in the form of pyridoxine was assessed in 60 patients and its efficacy was reported in 20 (21.1%). Median VB6 dosage was 10 mg/kg/die (8.5–10.0).

### End stage kidney disease and transplantation

**Fig. 1:**
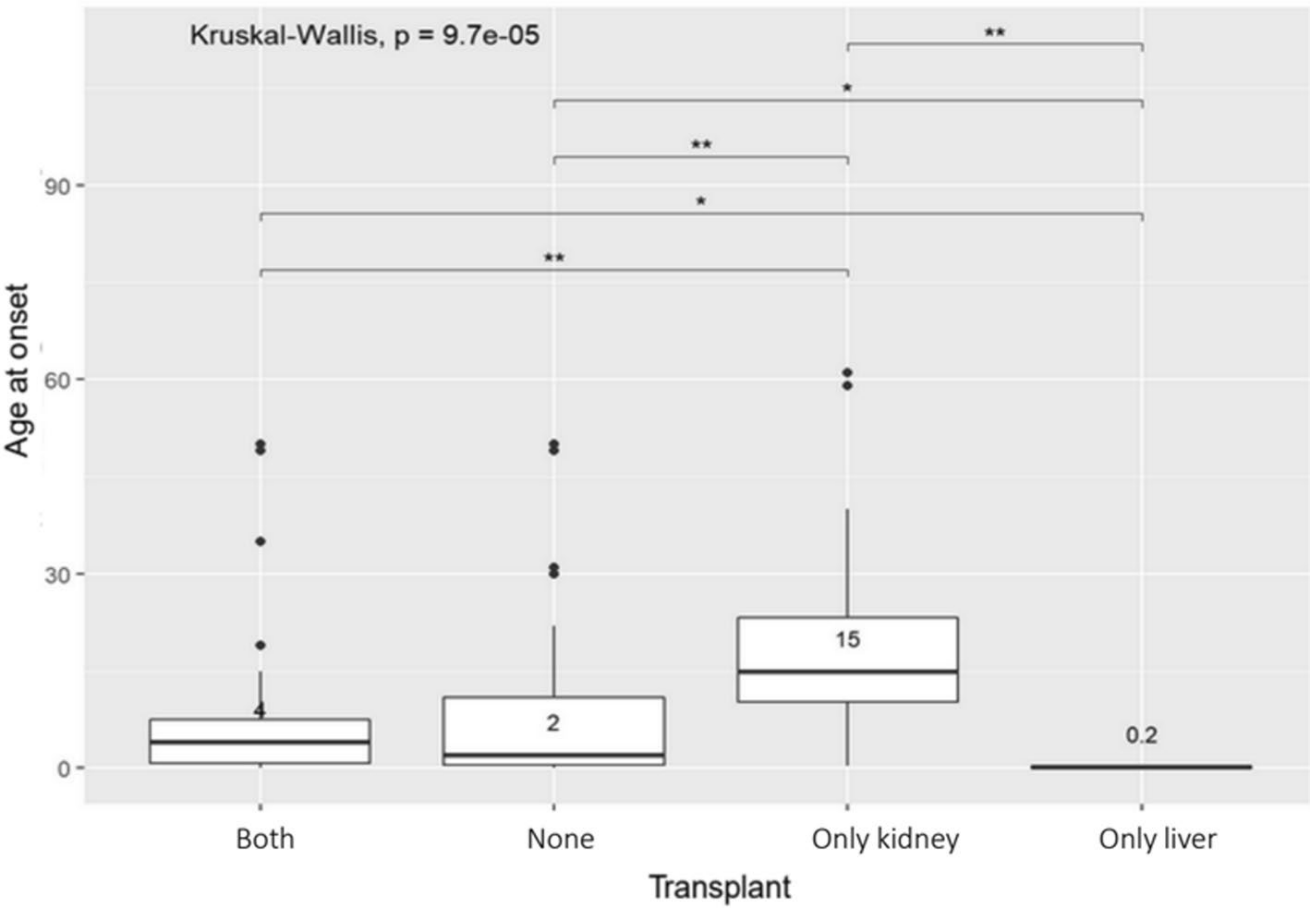
Box plot of age at onset of symptoms in patients receiving kidney and liver transplant, no transplantation, only kidney transplant, only liver transplant

At the end of June 2020, 63 patients (66.3%) had developed ESKD. Median age at ESKD was 14.0 years (5.0–31.5). No differences between males and females were observed. Twenty-one patients had a kidney-only transplant and, among them, four had a second kidney-only transplant. In second other cases, the second kidney transplant was combined with liver transplant and one of the patients received two further kidney transplants after their liver transplant; in one case liver transplant was performed together with the third kidney transplant. Median age at first kidney transplant was 16.5 years (9.3–36.3); patients receiving a kidney-only transplant tended to have a higher age at onset compared to those receiving a combined kidney–liver or liver-only transplant (Fig. 1). Combined kidney–liver transplant was performed in 29 patients, while two patients underwent a sequential kidney–liver transplant. Median age at kidney–liver transplant was 12 years (4.0–27.0). In 3 cases a second kidney transplant was performed after the combined kidney–liver transplant, and one patient received a second liver transplant. In five cases a preemptive liver transplant was performed. Those receiving a liver-only transplant tended to have lower renal function at last follow-up.

### Genotype–phenotype correlations

Most of the PH patients in our large Italian cohort came from southern Italy and the islands. As reported in the literature, the most common mutation, even in the Italian cohort, was p.Gly170Arg (28% of the alleles), in 16 patients in homozygosis, in 14 in heterozygosis with a missense mutation, and in 8 with a null mutation. Similar to literature data reported in other populations, the second most frequent mutation was the null c.33dupC mutation. Some mutations were recurrent among apparently unrelated patients coming from the same geographic area. The p.Gly156Arg and p.Ile244Thr mutations were only seen in patients from southern Italy and the islands, therefore, a founder effect or a genetic drift may be hypothesized for these mutations.

*AGXT* haplotype was consistent with published data in all cases [24].

**Fig. 2:**
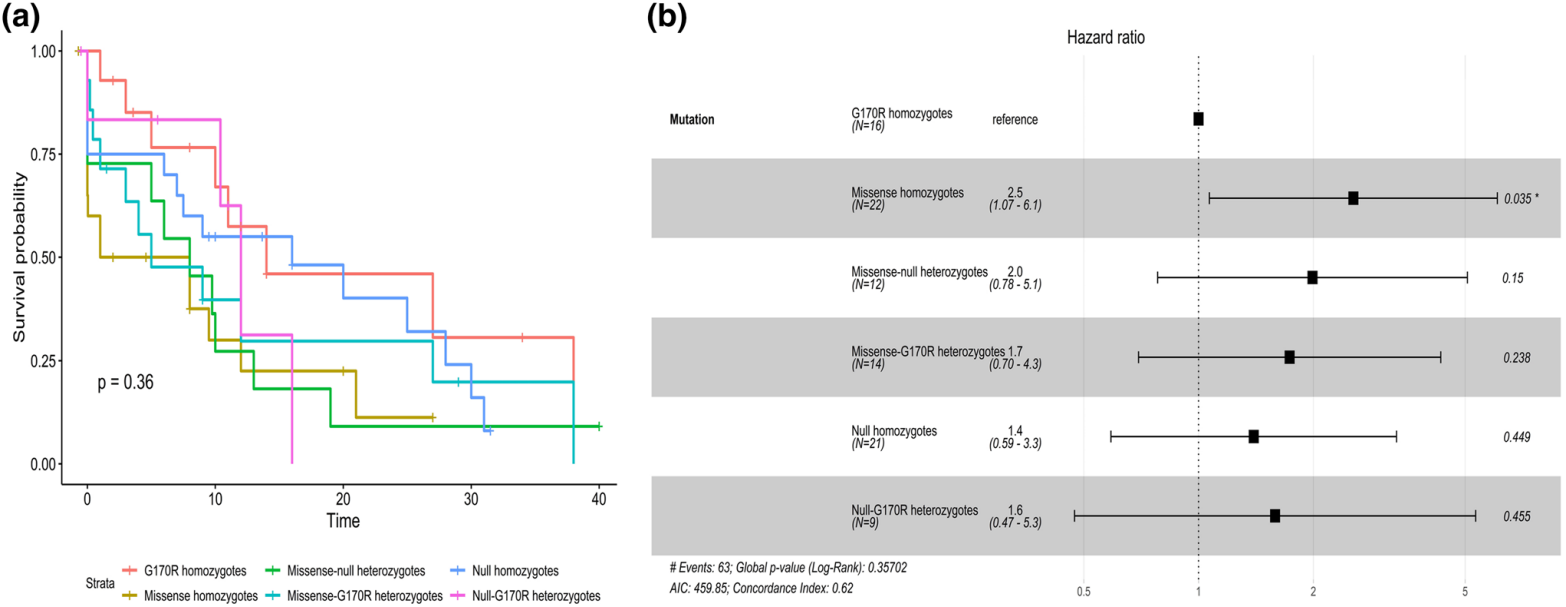
Kaplan–Meier curve (**a**) and HR plot (**b**) of ERKD per type of *AGXT* mutation (G170R homozygotes in red, missense homozygotes in golden, null homozygotes in dark blue, missense-null heterozygotes in green, missense-G170R heterozygotes in light blue, null-G170R heterozygotes in pink) (colour figure online)

**Fig. 3:**
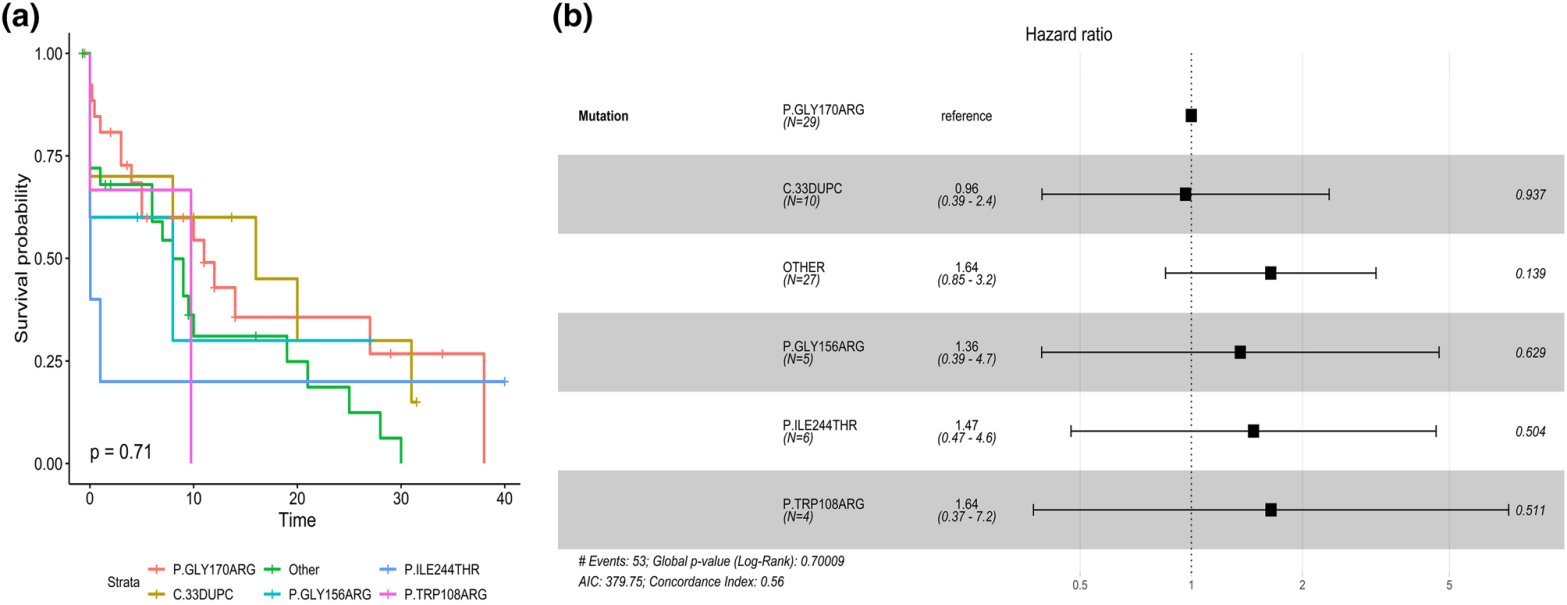
Kaplan–Meier curve (**a**) and HR plot (**b**) of ESKD per most frequent mutations of AGXT in the PH1 cohort

As expected, homozygotes for the common G170R mutation have the oldest age at onset of symptoms (17 years; 6.0–23.5, p 0.035) and of ESKD (28.5 years; 22.3–44.3, p 0.059) (Fig. 2). G170R homozygotes tend to have a lower prevalence of nephrocalcinosis at diagnosis, as expected. Kidney-only transplant is more frequent among patients carrying at least one G170R mutation (five homozygotes and four heterozygotes). Patients carrying the c.33dupC mutation (seven homozygotes and three compound heterozygotes) tend to have the youngest age at onset and the lowest eGFR at last follow-up, with the highest risk of ESKD (Fig. 3), as observed in a larger cohort [23].

## Conclusion

Here we present the first description of the largest cohort of Italian Primary hyperoxaluria patients collected over 30 years, contributing to delineate the genotypic and phenotypic spectrum of PH in different populations. The clinical characteristics of our cohort, as expected, largely overlap with those published by the OxalEurope consortium [23] in terms of age at onset of symptoms (3.9 years vs 4.0 years) and median age at diagnosis (8.1 years vs 9.9 years), slightly younger than age at onset (5.2 years) reported by the Rare Kidney Stone Consortium [1]. However, we observed a slightly higher frequency of nephrocalcinosis as a symptom at onset (46.3% NL–33.7% NC vs 32% NL–17% NC), in line with what was reported by the Rare Kidney Stone Consortium (30.6% NC) (Table 1).

It is noteworthy that 56% of our patients were diagnosed only after renal failure, and that the time from symptoms onset to PH diagnosis is still very long (median 3.2 years), although a trend to a more timely diagnosis has been observed in recent years. Moreover, the number of PH2 and PH3 patients in our country was low, thus suggesting a missed diagnosis of these rare diseases, especially in adult patients.

On the basis of exome sequencing data in the general population, Hopp et al. [3] estimated a carrier frequency for PH2 and PH3 of 1:279 and 1:185, with an expected prevalence of the disease of 1:310,075 and 1:135,866, respectively. This demonstrates that PHs in general, but PH2 and PH3 in particular, are highly underdiagnosed in Italy, most likely due to the milder course of the disease and the less probable evolution to ESKD. Therefore, it is crucial to increase the clinician’s awareness of these rare diseases; metabolic screening for all children at first presentation of a kidney stone or nephrocalcinosis, and for all adults with recurrent calcium oxalate kidney stones, could guarantee the best clinical management, as early conservative treatment may delay renal damage, particularly in pyridoxine responsive patients.

Regarding the genotype–phenotype correlation of *AGXT* mutation classes, we confirmed the better clinical course of patients carrying the G170R mutation. In our population, G170R homozygotes had the oldest age at onset of symptoms (17.0 years; 6.0–23.5, p 0.035) and of ESKD (28.5 years; 22.3–44.3, p 0.059). Interestingly, the median age at onset in our cohort is considerably higher than what is reported by the OxalEurope consortium (6 years), while the age at ESKD onset did not differ substantially (28.5 years vs 33.9 years), although our cohort is affected by a low number of patients. Genotype–phenotype comparison with the Rare Kidney Stone Consortium cohort [1] is not feasible because of different mutation grouping.

In line with previous reports involving European patients, we found several recurrent mutations in addition to G170R. Rumsby et al. demonstrated that Gly170Arg, c.33dupC and p.Ile244Thr are the most common mutations in Europe [25] and these data were confirmed by several other Authors [11, 26]. In agreement with these findings, the c.33dupC and the p.Ile244Thr mutations were found in 14% (27/190 alleles) and 5.7% (11/190 alleles) of patients in our cohort, respectively. The p.Ile244Thr mutation was found only in southern Italy and in the islands, as expected from its Spanish origin. (South Italy and the islands belonged to Spanish dominion during the Renaissance). In the same geographic areas, the p.Gly156Arg (12/190—6.3% of alleles) was also observed, at variance with other Italian regions, suggesting a founder effect.

Several of the mutations observed in the Italian cohort have been studied from a molecular perspective, such as the p.Trp108Arg on the mi allele, found in 6% of our patients (two homozygotes and four compound heterozygotes). Trp108 is located at the AGT active site and directly interacts with the coenzyme. The p.Trp108Arg variant shows strongly decreased catalytic activity and coenzyme binding affinity, two features suggesting a poor response to VB6 supplementation, as found in two of our patients [27]. Another example is represented by mutations affecting Ile56, the consequences of which, at the protein and cellular level, including in particular the response to Vitamin B6, are modulated by the allelic background, consistent with the clinical features of patients (5). These two studies have proved how clinical and molecular data can be combined to improve our comprehension of the phenotype and guide clinicians towards the best therapeutic strategy for PH1 patients.

Therefore, full *AGXT* molecular analysis with the reconstruction of the haplotype should always be reported, even when the mutations are found by multigene panel analysis or exome sequencing: it is extremely useful for the clinician because it could help in predicting the functional defect of the disease-causing mutations in synergy with the haplotype [28], thus guiding the therapeutic decision (e.g. VB6 supplementation) and predicting the clinical course. Even if PH eventually leads to ESKD, genotypes with significant residual enzymatic activity are associated, on average, with a longer disease-free period, and the same mutation in the background of different haplotypes could lead to different clinical courses [8]. Moreover, *AGXT* genotyping will be fundamental in order to have access to the new therapies developed in recent years, which are mainly focused on substrate reduction for the oxalate-producing enzymes using RNA-interference (RNAi) [29]. In November 2020 the first of the RNAi-based therapies was approved by EMA and the FDA (Lumasiran—Oxlumo^®^ that blocks the production of the enzyme glycolate oxidase, which is involved in the production of glyoxylate. https://www.ema.europa.eu/en/medicines/human/EPAR/oxlumohttps://www.fda.gov/news-events/press-announcements/fda-approves-first-drug-treat-rare-metabolic-disorder), while a second one (Nedosiran that inhibits production of the hepatic lactate dehydrogenase (LDH) enzyme, involved in the conversion of glyoxylate to oxalate) is currently being evaluated in clinical trials for all three types of PH (NCT04580420; NCT03847909; NCT04042402; NCT04555486, NCT05001269) [30]. Therefore, it is crucial for PH patients to be properly diagnosed and treated with the most appropriate therapy based on their individual mutations and clinical status [31].

Our study of PHs in Italy underlines the need for a more extensive use of both metabolic screening among patients with recurrent kidney stones and genotyping, including unambiguous assignment of minor/Major allele status. The considerable diagnostic delay, which has only slightly decreased in recent years, may partly explain the overall adverse outcomes in several patients. Limitations of this report include the absence or partial collection of some clinical data, such as details on stone episodes, on VB6 responsiveness and on biochemical dosages, and the lack of a systematic nationwide data collection, all of which hinder accurate prevalence and incidence estimates.

Considering some missing data for patients diagnosed by multigene next generation sequencing not referred to our Center and an Italian population of around 59.250.000 (Istat data https://www.istat.it/it/archivio/264511), we can hypothesize a PH prevalence of 1.4:1,000,000, in line with most clinical cohorts.

These limitations could be partly overcome by the participation in the OXALEurope database of PH patients, which will be able to provide a better understanding of the diseases in terms of clinical characteristics and prevalence. This would also be a useful tool to reach all PH patients, with important implications for their clinical management (a new therapy is already available for compassionate use in Italy).

Notwithstanding these limitations, our report clearly shows the relevance of this rare disease in Italy and the need to increase awareness of PH among clinicians, especially in the perspective of the new therapies that will be available in the near future.

## Supplementary Information

Below is the link to the electronic supplementary material.

Supplementary file1 (PDF 123 KB)
