# Supplementary material for: Primary hyperoxaluria in Italy: the past 30 years and the near future of a (not so) rare disease
Source: J Nephrol. 2022 Feb 26;35(3):841–50. doi: 10.1007/s40620-022-01258-4 (PMC8995259; doi:10.1007/s40620-022-01258-4)
Supplement: Supplementary file 1 — Supplementary file1 (PDF 123 KB) [file 40620_2022_1258_MOESM1_ESM.pdf]

Name	Institution	City
**Francesca Becherucci**	A.O. Universitaria Meyer	Firenze
**Bertuzzi Veronica**	Ospedale S. Maria della Misericordia	Urbino
**Tortora Giada**	Ospedali Riuniti	Ancona
**Cristina Malaventura**	Ospedale Ferrara	Ferrara
**Gabriele Donati**	AOU P. S. Orsola-Malpighi	Bologna
**Andrea Pasini**	AOU P. S. Orsola-Malpighi	Bologna
**Berardi Sonia**	AO Policlinico S. Orsola - Malpighi	Bologna
**Maggiore Umberto**	Azienda Ospedaliero-Universitaria di *Parma*	Parma
**Valeriana Colombo**	Ospedale Niguarda	Milano
**Lorenzo D'Antiga**	AO Papa Giovanni XXIII – Bergamo	Bergamo
**Delprete Dorella**	Azienda Ospedaliera di Padova - Dimed	Padova
**Marchini Francesco**	Azienda Ospedaliera di Padova	Padova
**Boero Roberto**	Ospedale Martini	Torino
**Tandoi Francesco**	Ospedale Molinette	Torino
**Cussa Davide**	Ospedale Molinette	Torino
**Biancone Luigi**	Ospedale Molinette	Torino
**Tognarelli Giuliana**	AOU S. Luigi Gonzaga	Orbassano (TO)
**Chiarinotti Doriana**	AOU Maggiore della Carità	Novara
**Diego Bellino**	Ospedale S. Martino	Genova
**Gian Marco Ghiggeri**	IRCCS IP Giannina Gaslini	Genova
**Paglialonga Fabio**	IRCCS Cà Granda Ospedale Maggiore Polilinico Milano	Milano
**Taroni Francesca**	IRCCS Cà Granda Ospedale Maggiore Polilinico Milano	Milano
**Gentile Anna**	Ospedale di Trento	Trento
**Di Palma Teresa**	Ospedale di Trento	Trento
**Tubaro Martina**	Pediatria Ospedale Santa Maria degli angeli	Pordenone
**Monardo Paolo**	Ospedale Papardo	Messina
**Mario Giordano**	Ospedale Policlinico di Bari	Bari
**Diletta Torres**	Ospedale Policlinico di Bari	Bari
**Aceto Gabriella**	Ospedale Policlinico di Bari	Bari
**Malgieri Gabriele**	Ospedale Santobono di Napoli	Napoli
**Miriam Zacchia**	AOU Università degli Studi della Campania "Luigi Vanvitelli"	Napoli
**Ferretti Alfonso**	Ospedale Santobono	Napoli
**Salvatore Coppola**	UOSD Nefrologia e dialisi, P.O. Piedimonte Matese	Caserta
**Cuomo Vincenzo**	UOSD Nefrologia e dialisi, P.O. Piedimonte Matese	Caserta
**Catalano Francesco**	Ospedale Bianchi Melacrino Morelli	Reggio Calabria
